# Heterostylous plants in an era of global change: a review on the consequences of habitat loss and fragmentation

**DOI:** 10.1093/aobpla/plaf016

**Published:** 2025-03-22

**Authors:** Marianne Kivastik, Sílvia Castro, Elena Conti, Hans Jacquemyn, Barbara Keller, Attila Lengyel, Michael Lenhard, Zuzana Münzbergová, Iris Reinula, Bojana Stojanova, Sabrina Träger, Mari-Liis Viljur, Tsipe Aavik

**Affiliations:** Institute of Ecology and Earth Sciences, University of Tartu, J. Liivi 2, 50409 Tartu, Estonia; Centre for Functional Ecology – Science for People & the Planet, Department of Life Sciences, University of Coimbra, Calçada Martim de Freitas s/n, 3000-456 Coimbra, Portugal; Department of Systematic and Evolutionary Botany, University of Zürich, Zollikerstrasse 107, 8008 Zürich, Switzerland; Department of Biology, Plant Conservation and Population Biology, Katholieke Universiteit Leuven, Kasteelpark Arenberg 31, 3001 Leuven, Belgium; Department of Systematic and Evolutionary Botany, University of Zürich, Zollikerstrasse 107, 8008 Zürich, Switzerland; Institute of Ecology and Botany, HUN-RENCentre for Ecological Research, Alkotmány u. 2-4., 2163 Vácrátót, Hungary; HUN-REN-EKKE Lendület Environmental Microbiome Research Group, Leányka u. 6, H-3300 Eger, Hungary; Institute for Biochemistry and Biology, University of Potsdam, Karl-Liebknecht-Str. 24-25, D-14476 Potsdam-Golm, Germany; Department of Botany, Faculty of Science, Charles University, Albertov 2038/6, 128 00 Prague, Czech Republic; Institute of Botany, Czech Academy of Sciences, Zámek 1, 252 43 Průhonice, Czech Republic; Institute of Ecology and Earth Sciences, University of Tartu, J. Liivi 2, 50409 Tartu, Estonia; Department of Biology, University of Naples Federico II, Complesso Universitario MSA, Naples, Italy; Institute of Biology/Geobotany and Botanical Garden, Martin Luther University Halle-Wittenberg, Große Steinstraße 79/80, 06108 Halle (Saale), Germany; German Centre for Integrative Biodiversity Research (iDiv) Halle-Jena-Leipzig, Puschstraße 4, 04103 Leipzig, Germany; Institute of Ecology and Earth Sciences, University of Tartu, J. Liivi 2, 50409 Tartu, Estonia; Institute of Ecology and Earth Sciences, University of Tartu, J. Liivi 2, 50409 Tartu, Estonia

**Keywords:** heterostyly, habitat loss, habitat fragmentation, animal pollination, gene flow, homostyly, morph ratio, population size

## Abstract

The widespread loss and fragmentation of habitats have caused significant declines in biodiversity. Among plants, animal-pollinated species are particularly threatened because of the negative effects of these factors on pollinators. Heterostyly is a unique reproductive system defined by two or three floral morphs having a distinct position of anthers and style. The spatial separation of reproductive organs, accompanied by a self-incompatibility system, restricts self-pollination and favours pollinator-mediated pollen transfer between different morphs. In this review, we synthesize knowledge about the effects of loss in the area and connectivity of habitats, and related reduction in population size on heterostylous plants. We conducted a literature search to obtain an overview of studies investigating the short- and long-term consequences of the decreased area and connectivity of habitats as well as plant population size for heterostylous species. To quantify the relationship between plant population size and morph ratio bias, we applied a meta-analytical approach. First, the meta-analysis showed that reductions in population size can significantly disrupt the optimal morph ratio, leading to fewer compatible mates and lower reproductive output. Second, the literature review highlights the negative consequences of biased morph ratios for population viability and genetic diversity of heterostylous plants. Finally, heterostylous species may adapt to the loss of pollination by shifting their mating system to homostyly and selfing. This review demonstrates that habitat loss and fragmentation have various consequences for heterostylous plants, e.g. reduced population size, morph ratio bias, and disruption of pollination. With ongoing environmental changes, there are still important knowledge gaps that need to be addressed more systematically. These include the long-term impact of skewed morph frequencies on population viability, the selective forces driving variation in anther-stigma separation and intra-morph compatibility, the role of habitat loss and connectivity, and related reduction in pollinator abundance and diversity in the selection for homostyly.

## Teaser

This paper examines the impact of habitat loss and fragmentation on heterostylous plants, which rely on pollinators for successful reproduction. By synthesizing existing research and conducting a meta-analysis, the study reveals that population size reductions can lead to morph ratio biases, resulting in fewer compatible mates and lower reproductive success. These imbalances threaten population viability and genetic diversity and potentially further contribute to evolutionary changes. This paper demonstrates the vulnerability of heterostylous plants to landscape changes and emphasizes the need to better protect these species and their habitats for the well-being of other animal-pollinated species as well.

## Introduction: landscape change effects on biodiversity

Human-induced landscape changes, resulting in the loss and fragmentation of habitats, are among the main threats to plant diversity (Haddad *et al.* 2015; IPBES 2019). Habitat fragmentation reduces the size of habitats and increases isolation between fragments. Plant populations in fragmented habitats experience a decrease in size and reduced functional connectivity (i.e. seed and pollen flow), which can eventually lead to impoverished within-population genetic diversity (Leimu *et al.* 2010) and lower reproductive output (Cranmer *et al.* 2012; Mendes *et al.* 2022). The impact of habitat loss and decreased connectivity on species depends on different life history traits, such as mating and compatibility systems, pollination and seed dispersal mode, and life span (Aguilar *et al.* 2008; Marini *et al.* 2012; Moser *et al.* 2015). A vast majority of flowering plants (from 78% in the temperate zone up to 94% in tropical regions) rely on animal-mediated pollination (Ollerton *et al.* 2011; Tong *et al.* 2023). Recent studies have shown a drastic loss in insect diversity and abundance (Hallmann *et al.* 2017; Powney *et al.* 2019), with severe negative consequences for plant-pollinator interactions. The significant decline in pollinators poses a threat to the reproduction of numerous plant species (Rodger *et al.* 2021; Artamendi *et al.* 2024) and may lead to the disappearance of species that rely on cross-pollination by insects (Biesmeijer *et al.* 2006). Therefore, self-incompatible animal-pollinated species and species with complex mating systems, such as heterostyly, are considered to be particularly vulnerable (Aguilar *et al.* 2006). In contrast, plants capable of autonomous self-pollination are at lower risk, even in fragmented habitats, as they are not so dependent on pollinators for reproduction (Honnay and Jacquemyn 2007; Aguilar *et al.* 2008).

**Figure 1.**
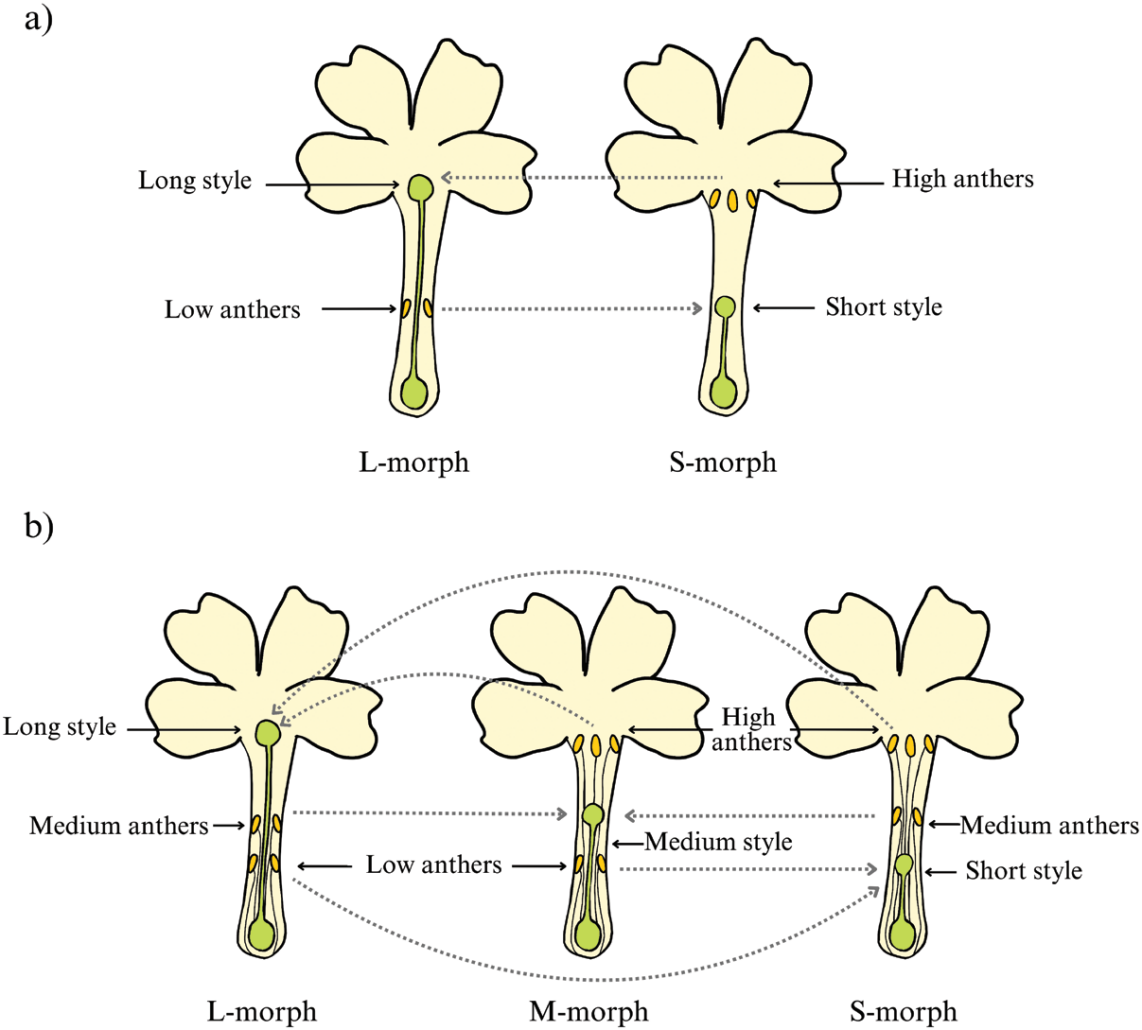
A schemae of heterostylous plants representing the morphologies and compatibilities between different morphs for (a) distylous plants (two morphs based on *Primula* sp.) and (b) tristylous plants (three morphs based on *Oxalis* sp.). The dashed line represents the direction of compatible pollination, leading to successful fertilization.

Heterostyly, a floral polymorphism observed in at least 28 angiosperm families (Naiki 2012), is characterized by the presence of two (distyly) or three (tristyly) genetically determined floral morphs (Fig. 1), each with reciprocal positioning of sexual organs (Darwin 1877; Barrett 2019). Although some authors consider heterostylous and other style-length polymorphic taxa together in their studies, thus updating the number of families to 34 (Simón-Porcar *et al.* 2024), here, our focus is specifically on heterostylous taxa. Darwin, being the first to suggest an explanation for this floral polymorphism, proposed that it promotes cross-fertilization between individuals with different floral morphologies (Darwin 1862, 1877). Distylous species comprise long-styled morphs (L-morph or pin) and short-styled morphs (S-morph or thrum; Fig. 1a). In the S-morph, the style is short and positioned lower in the corolla tube, while anthers are located at a higher position. In the L-morph, the style is long, while the anthers are placed lower inside the corolla. Tristyly is characterised by the presence of a third floral form—the mid-styled morph (M-morph), with the style in the middle, and anthers located both at lower and higher positions relative to the style (Fig. 1b). Such a matching spatial arrangement of sexual organs is known as reciprocal herkogamy and promotes disassortative pollination through differential pollen placement onto different parts of the pollinator’s body (Webb and Lloyd 1986; Keller *et al.* 2014; Trevizan *et al.* 2021).

**Figure 2.**
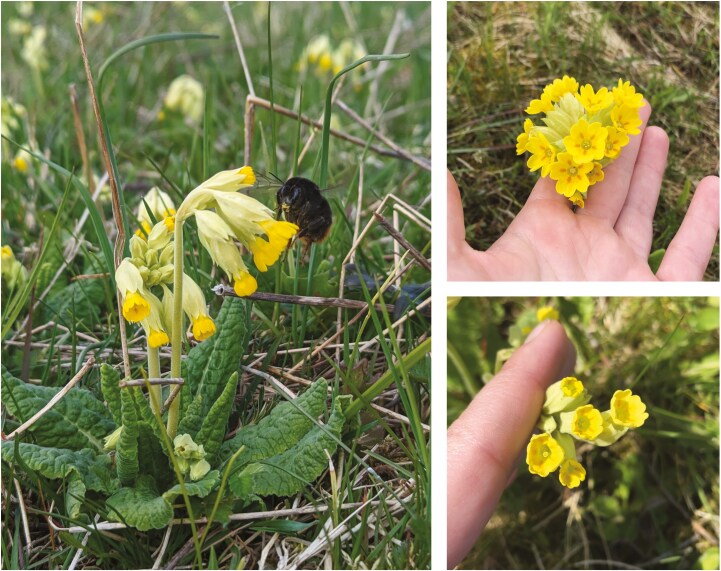
*Primula veris* is one the most studied heterostylous species and is often used as a model species representing the heterostyly system. The different floral morphs are visibly recognized by looking inside the flower. The S-morph (anthers visible) is on the upper right picture, and the L-morph (stigma visible) is on the lower right.

Heterostylous plants are usually perennials with medium-sized, tubular-shaped, animal-pollinated flowers (Barrett 1992; Fig. 2). The reciprocal positioning of sexual organs in heterostylous species is generally accompanied by a heteromorphic incompatibility system. This system prevents the pollen from the same floral morph from germinating on the stigma, ensuring optimal fertilisation between different floral morphs and very low or non-existent seed production after intra-morph pollination. Therefore, in equilibrium, heterostylous plant populations comprise equal frequencies of each floral morph (i.e. isoplethy) through negative frequency-dependent selection and disassortative pollination, ensuring maximum reproductive output. The degree of self-incompatibility is, however, quite variable between different plant families, species and even between populations, individuals, and different morphs within the same species (Wedderburn and Richards 1990; Zietsman *et al.* 2008; Shao *et al.* 2019).

Heterostyly is determined by a heterostyly supergene or *S*-locus, which is a chromosomal region containing several tightly linked genes that determine different heterostyly traits, such as the length of the style and stamens, self-incompatibility, and the size of pollen grains (Huu *et al.* 2016; Li *et al.* 2016). Recent molecular studies in *Primula* have determined that the *S*-locus is hemizygous, being present only in the dominant *S* haplotype (S-morph) and absent in the recessive *s* haplotype (L-morph) (Kappel *et al.* 2017; Potente *et al.* 2022). The same pattern has been confirmed in *Turnera* (Shore *et al.* 2019), *Linum* (Gutiérrez-Valencia *et al.* 2022) and *Fagopyrum* (Fawcett *et al.* 2023). Mutations in the *S*-locus have been associated with the breakdown of heterostyly and changes in self-compatibility (Kappel *et al.* 2017).

**Figure 3.**
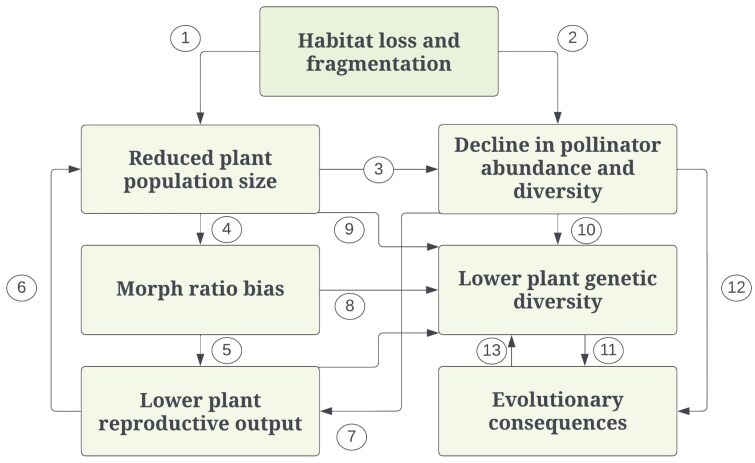
Potential consequences of habitat loss and fragmentation for heterostylous species. The main direct consequence of habitat loss and fragmentation is a reduction in population size (1). Since heterostylous plants depend on animal pollination, their reproductive success is directly affected by the availability and diversity of pollinators, which can be reduced as a result of habitat fragmentation (2). Smaller plant populations also support fewer pollinators (3). A reduction in population size may lead to skewed morph ratios, leading to fewer suitable mates for reproduction (4). This imbalance in morph ratios leads to lower reproductive output through a reduction in seed production (5), which in turn leads to smaller population sizes (6). Pollinator abundance and diversity have a direct effect on the reproductive output of heterostylous populations by reducing pollen dispersal (7). Morph ratio bias and smaller population size can cause more (biparental) inbreeding and thus negatively affect the genetic diversity of populations (8, 9). With fewer pollinators, there is less movement among individuals inside and between populations and thus less gene flow, directly affecting genetic diversity (10). Populations with lower genetic diversity have lower adaptive evolutionary potential to respond to environmental change (11). A decline in pollinators can lead to a shift in the mating system by evolving towards self-compatibility and homostyly or, alternatively, by becoming more visible and attractive to pollinators (12). Evolution to self-compatibility can lead to more inbreeding and reduced genetic diversity (13).

Land use changes that lead to a decline in pollinator populations have detrimental effects on plant species with complex reproductive systems, specialized pollination, and elaborate floral architecture (Stefanaki *et al.* 2015). Heterostylous plants rely on pollinators to deposit the pollen on their bodies to transfer it to the compatible morph type’s stigma (Brys and Jacquemyn 2020). Due to their dependence on the availability of pollinators for successful reproduction, heterostylous plants fall into the group of species that are particularly vulnerable to the recent loss and fragmentation of natural and semi-natural habitats. Due to animal pollination and various other traits unique to heterostylous species, such as disassortative intermorph mating, equal morph frequencies, and self-incompatibility, fragmentation poses several threats to the short-term fitness and long-term viability of heterostylous plants (Fig. 3). Habitat loss and fragmentation negatively affect the abundance, community composition and movement of pollinators (Matsumura and Washitani 2000; Waites and Ågren 2004; Ishihama and Washitani 2007; Van Rossum *et al.* 2015), which, in turn, may translate into reduced fitness of heterostylous plant populations in the short term (Kang *et al.* 2000) or initiate evolutionary shifts of mating system (Yuan *et al.* 2017). The negative effects of pollinator loss are especially pronounced in heterostylous plants due to their self-incompatible mating system. Habitat loss also imposes direct effects on the populations of heterostylous plants through reduced population sizes leading to unbalanced morph ratios (Endels *et al.* 2002; Kéry *et al.* 2003; Aavik *et al.* 2020), which, in turn, can intensify the loss of genetic diversity (Van Rossum and Triest 2006a; Meeus *et al.* 2012; Kaldra *et al.* 2023). This limits mating opportunities more strongly in heterostylous species than in monomorphic self-incompatible species. Despite the possible vulnerability of heterostylous plants to habitat fragmentation, we lack a systematic understanding of the various consequences of landscape change and related habitat loss on these species. A comprehensive overview is thus crucial not only for proposing effective conservation measures to secure the persistence of these plants, but also for highlighting gaps in knowledge about the fascinating reproductive system of heterostyly (Barrett 2019).

In this review, we provide a systematic overview of the different mechanisms by which habitat fragmentation can influence heterostylous plants through changes in mating patterns. First, we conducted a thorough review of the scientific literature to extract case studies that explore the short- and long-term response of various aspects of habitat fragmentation on heterostylous species and their fitness. Second, we performed a meta-analysis to quantify the relationship between population size and morph ratios. We synthesize information from different heterostylous species to obtain an improved understanding of the impact of habitat fragmentation. Given that several heterostylous species are already highly endangered and need better conservation practices (Yamamoto *et al.* 2017), we provide recommendations to guide the development of effective conservation measures and to predict the viability of these species in an era of global change. Finally, we identify the main gaps in our current knowledge about the consequences of fragmentation for plants with heterostylous mating systems.

## Methods

### Literature search

We conducted a keyword search on Web of Science (https://www.webofscience.com/) to get an overview of the scientific literature on the effects of fragmentation on heterostylous species. We used keyword combinations of heterostyly / distyly / tristyly / heteromorphic incompatibility / fragmentation / population size / habitat loss / connectivity / morph ratio / morph bias. Heterostyly, distyly, tristyly, and fragmentation keywords were searched as heterostyl*, distyl*, tristyl* and fragment* to consider all possible forms such as ‘heterostyly’, ‘heterostylous’, ‘heterostyle’ as well as ‘fragmented’, ‘fragmentation’ and so on. Heterostyl* + ‘morph ratio’ combination resulted in most results (41 unique articles) and other combinations of searches returned largely the same articles. The least effective combinations were with heteromorphic incompatibility, which yielded at most five results with a combination of population size, that were already present in previous searches as well, indicating that the term ‘heteromorphic incompatibility’ is least often used as a keyword. As of October 21, 2024, our search yielded a total of 149 unique articles. We conducted the PRISMA protocol (Page *et al.* 2021) on the literature search results to identify suitable studies (See Supplementary materials for a detailed flow diagram and descriptions, and the table with a list of search results). All search results were initially screened by the titles and abstracts before a more detailed evaluation. This screening excluded records that were:

(1) not written in English (only English abstracts available);
(2) the word string led to a different meaning. For example, for the keyword ‘fragment*’, a few articles discussed DNA fragments rather than habitat fragments;
(3) papers were of other (polymorphic) species and not heterostylous species (e.g. style-dimorphic *Narcissus* species).

This screening resulted in the exclusion of 34 articles. All remaining 115 articles were individually checked for relevance. Here, articles were excluded when they were not suitable content-wise, such as:

(1) broad review articles;
(2) method developments or computational models (e.g. microsatellite markers);
(3) otherwise not connected to the study question (e.g. descriptions of species in general or determination of heterostyly in a particular species etc.).

This excluded a further 54 articles and resulted in a total of 61 articles that were found relevant to the study question posed here.

### Meta-analysis

Among the reviewed literature, we found that a considerable amount of studies reported a correlation between population size and morph ratios, for both distylous and tristylous species. We hence decided to conduct a meta-analysis of the available data to quantify the relationship between population size and morph ratio balance in addition to qualitatively reviewing and interpreting these observations. We checked the results of the literature search for studies that had recorded both population size and morph frequencies. Unfortunately, while in many studies morph ratios were recorded, corresponding sizes of the observed populations were reported with much lower frequency. Relevant data were extracted from 20 studies focusing on distylous species and 8 studies on tristylous species. We also tried to directly contact authors who had mentioned recording both morph frequencies and population sizes but did not either analyse it or have data available. For one study that was published over 20 years ago, the authors did not have access to the data anymore. Some relevant studies also appeared to have used the same datasets for many articles, so only one study from the same dataset was included. Data from one study that was not yet published at the time when the literature search was done (Aavik *et al.* 2025) was also included. Three studies had multiple recordings through different years, which were treated as separate observations. Indeed, 28 is not an especially representative amount of studies to conduct a meta-analysis on, but at the same time, this represents the current state of studies available and the fact that, although rather central and important aspects, the population size and morph ratio are not often recorded at the same time.

In the case studies of distylous plants, morph ratios were assessed by calculating the absolute value of the difference in the number of individuals of the long- and short-styled morphs divided by the total number of flowering individuals (S − L)/(S + L). For tristylous species, most studies tested the relationship between population size and the evenness of morph types. The morph evenness index (sensu Da Cunha *et al.* 2014) indicates how equally the different morphs are distributed in a population. We used Fisher’s *z*-transformed correlation coefficients to compare the correlations reported in different studies. The correlation coefficients and sample sizes were extracted from published texts. In studies where Pearson’s correlation coefficient (*r*) was not reported, we either calculated it from other published statistics or from the raw data available in tables or figures (we used https://automeris.io/WebPlotDigitizer for importing values from figures). When population size was reported as a categorical variable, we used Spearman’s rank correlation analysis and further transformed Spearman’s rho to Pearson’s *r* (based on Lajeunesse 2013). We then calculated effect sizes and corresponding sampling variances using the escalc function with ‘ZCOR’ measure in the metafor package. We fitted two random-effects meta-analysis models on the data of (1) distylous plants and (2) tristylous plants. We used the rma.mv function in the metafor package for building the models and assessing the heterogeneity among studies (Viechtbauer 2010). The identity of a case study was included as a random factor in the models to account for dependent effect sizes arising from multiple correlation coefficients within the same study. For ease of interpretation, we back-transformed Fisher’s *z* to Pearson’s *r* after the analysis. All analyses were conducted in R 4.3.1 (R Core Team 2023).

## The response of heterostylous plants to loss in the area and connectivity of habitats

### The effect of population size on morph ratios

**Figure 4.**
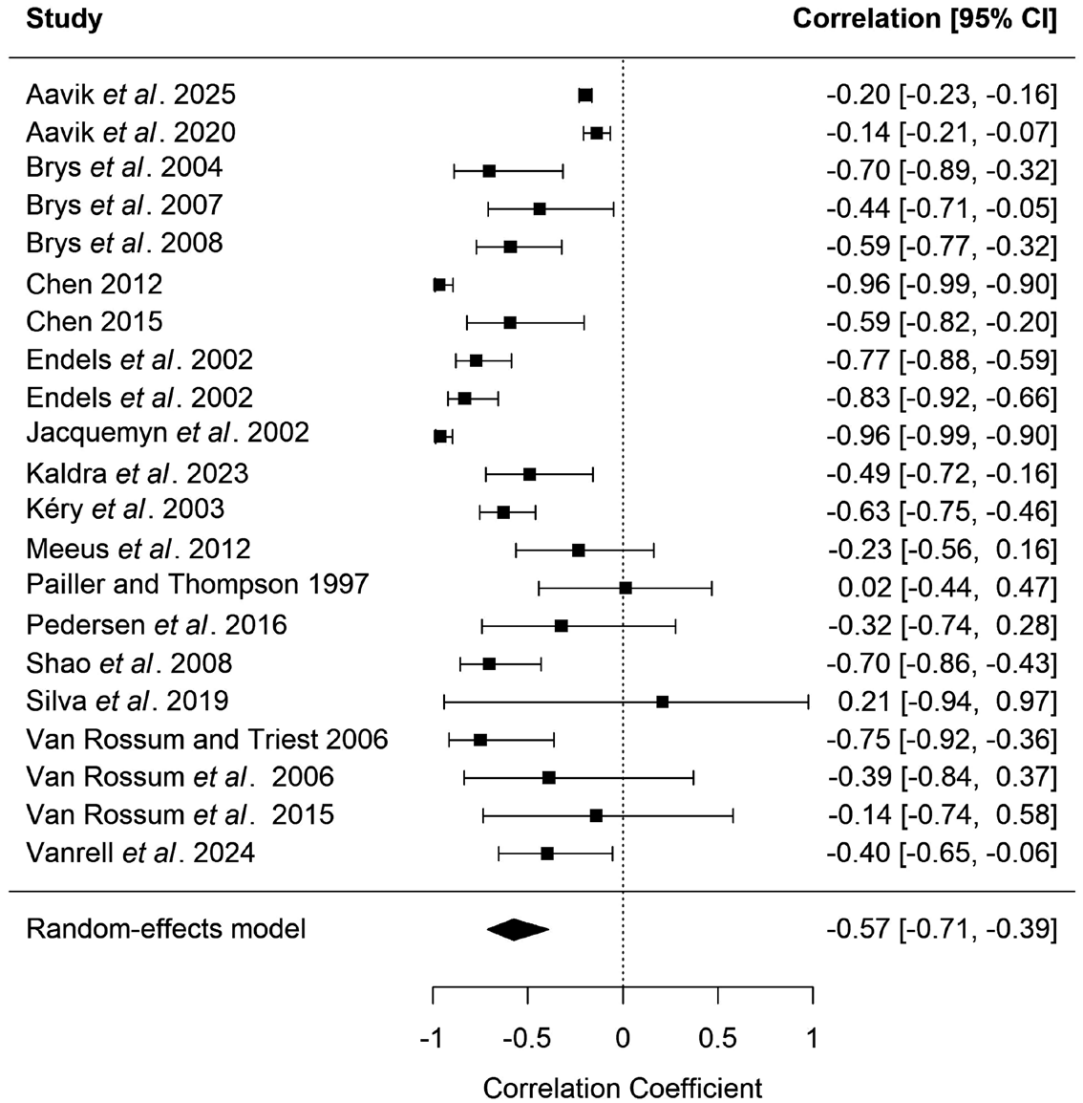
Results of the meta-analysis investigating the correlation between plant population size and morph ratio bias based on results from studies on distylous species. The study by Endels *et al.* 2002 had two recordings through different years, which were treated as separate observations. Pearson correlation coefficients with 95% confidence intervals are shown. The diamond represents the mean correlation coefficient estimated from the model.

**Figure 5.**
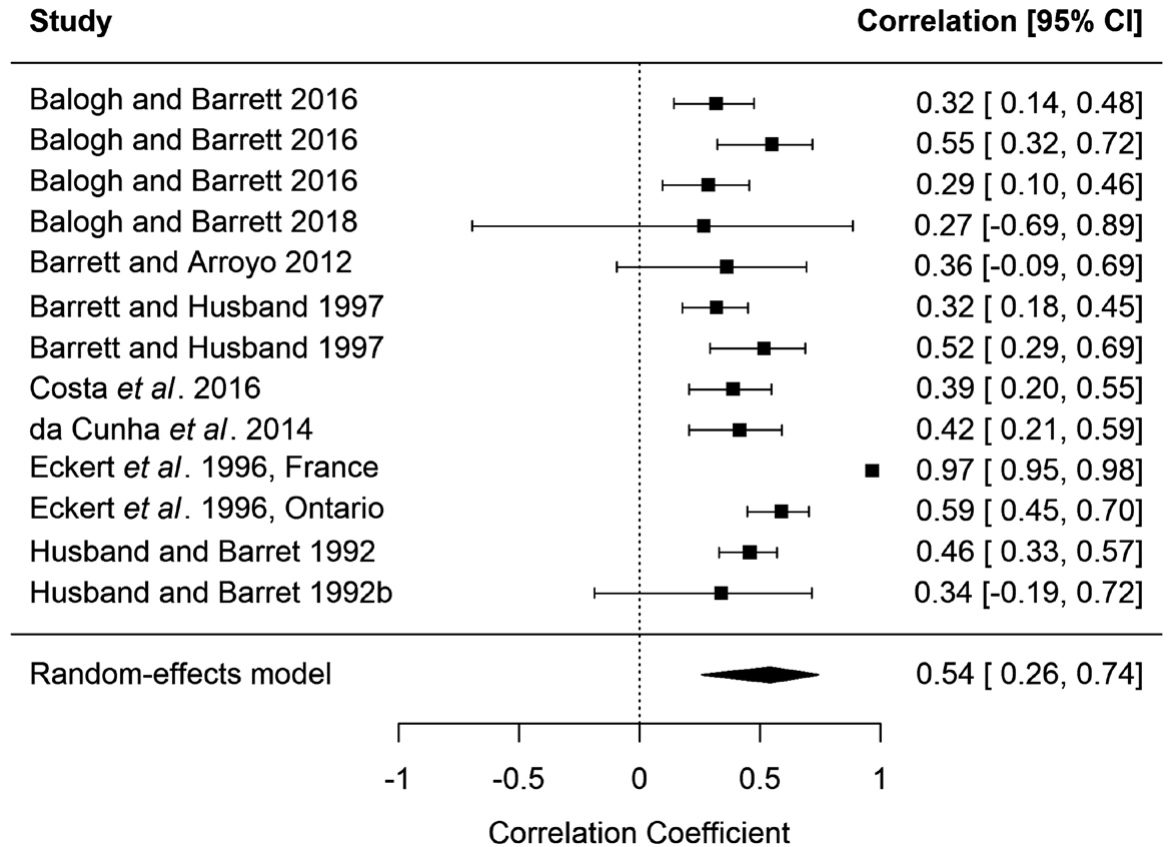
Results of the meta-analysis investigating the correlation between plant population size and morph evenness based on the results from studies on tristylous species. The studies by Balogh and Barrett 2016 and Barrett and Husband 1997 had multiple recordings through different years, which were treated as separate observations. Pearson correlation coefficients with 95% confidence intervals are shown. The diamond represents the mean correlation coefficient estimated from the model.

The random-effects meta-analysis showed a significant negative correlation between population size and morph ratio bias in distylous plant species (*r* = −0.57, 95% *CI* [−0.71, −0.39], *p* < 0.001, Fig. 4). Similarly, larger populations of tristylous species had higher evenness of different flower morphs (*r* = 0.54, 95% *CI* [0.26, 0.74], *p* < 0.001, Fig. 5). The heterogeneity across studies was very high in both models (*Q* = 186.91, *df* = 20, *P* < 0.001, *I*^*2*^ = 89.30 in the model of distylous species and *Q* = 244.79, *df *= 12, *P* < 0.001, *I*^*2*^ = 95.10 in the model of tristylous species) implying that the effect size varied considerably among studies (see also Fig. 4 for across-study variation). While natural processes can lead to skewed morph ratios in small populations of heterostylous species, recent studies have indicated increased occurrence of morph biases as a result of human-induced habitat fragmentation, leading to the collapse of plant populations (Kéry *et al.* 2003; Aavik *et al.* 2020). At equilibrium, heterostylous plant populations are expected to maintain equal morph frequencies (i.e. isoplethy). For distylous species, the S- and L-morph ratio would be 1:1, and for tristylous 1:1:1 of S-, M-, and L-morph. Equal morph frequencies are maintained through negative frequency-dependent selection and disassortative pollination. A reduction in population size is one of the main factors affecting the optimal morph balance in populations of heterostylous plants (Husband and Barrett 1992a; Barrett and Husband 1997; Baker *et al.* 2000; Endels *et al.* 2002; Jacquemyn *et al.* 2002; Kéry *et al.* 2003; Van Rossum and Triest 2006a; Brys *et al.* 2008; Shao *et al.* 2008; Barrett and Arroyo 2012; Chen 2012; Da Cunha *et al.* 2014; Chen *et al.* 2015; Costa *et al.* 2016; Pedersen *et al.* 2016; Balogh and Barrett 2018; Aavik *et al.* 2020; Kaldra *et al.* 2023; Vanrell *et al.* 2024; Aavik *et al.* 2025). An abrupt decline in the abundance of individuals can result in a stochastic reduction in the proportion of any morph type, which is supported by the meta-analysis of case studies in both distylous and tristylous species (Figs. 4 and 5). However, not all reviewed studies reported significant associations between population size and morph ratios (Pailler and Thompson 1997; Lienert and Fischer 2003; Silva *et al.* 2019). For example, morph ratios in *Primula farinosa* were not affected by population size, most likely because all populations were large enough to buffer against demographic stochastic effects (Lienert and Fischer 2003).

Biased morph ratios affect the fitness of heterostylous plants in various ways. For instance, there is more outcrossing in larger populations compared to smaller populations, where the scarcity of mates can cause increased selfing, eventually leading to inbreeding depression (Ishihama *et al.* 2005; Van Rossum *et al.* 2006). The dominance of one morph over the other decreases the pool of suitable mates (lower effective population size, N_e_) and leads to fewer opportunities for reproduction (Kéry *et al.* 2003). In populations with skewed morph ratios, the negative consequences of fragmentation and the associated reduction in population size on fitness can be further amplified. Reproductive output is jeopardized not only by population size *per se* (Ågren 1996; Brys *et al.* 2004; Brys, Jacquemyn, De Bruyn, *et al.* 2007; Shao *et al.* 2008), but also through biased morph ratios. For example, in *Primula sieboldii*, biased morph ratios reduced population reproductive success by causing a significant decline in seed set (Ishihama *et al.* 2006). A study on *Pulmonaria officinalis* showed that seed set decreased significantly as the distance to a legitimate mating partner increased (Brys and Jacquemyn 2010). When morph frequencies were strongly biased, the more common morphs produced significantly fewer fruits and seeds per fruit (Brys *et al.* 2004), indicating negative frequency-dependent selection. However, mating among spatially close individuals (< 5 m) can lead to reduced progeny fitness due to the increased likelihood that these individuals are closely related (Ishihama *et al.* 2005). The negative impact of morph ratio bias on the fitness of the more frequent morph was also observed in populations of the distylous *Hottonia palustris* (Brys, Jacquemyn, and Hermy 2007). Similar trends have been simulated in computer models for tristylous species (Heuch 1979; Eckert *et al.* 1996).

The reduction in the abundance of one morph is expected to occur randomly; hence, morph bias can fluctuate towards either morph as a result of stochastic demographic processes. However, some studies indicate that a specific morph may become more dominant in smaller populations. Most of these studies indeed demonstrate that smaller populations show a higher prevalence of the L-morph (Chen 2012; Chen *et al.* 2015; Pedersen *et al.* 2016), though the opposite has also been detected (Brys *et al.* 2008). In tristylous populations, similar trends can be observed. For example, biased populations of *Lythrum salicaria* showed a lower frequency of the short-styled S-morph (Balogh and Barrett 2016; Costa *et al.* 2016). Similarly, in populations of *Eichhornia paniculata,* loss of the S-morph was observed to be the most frequent (Husband and Barrett 1992a). However, in the trimorphic populations of *Pontederia azurea* (formerly *Eichhornia*), the mid-styled morph was the least frequent (Da Cunha *et al.* 2014).

Several hypotheses have been proposed to explain the prevalence of a specific floral morph over other(s). It has been suggested that the behaviour of pollinators may affect the morph frequencies through variable visitation levels and distinct foraging patterns among morphs (Charlesworth 1979; Wolfe and Barrett 1987). However, a study on *Eichhornia paniculata* (Husband and Barrett 1992b) found no convincing evidence for this assumption. Another, more likely explanation for the prevalence of one floral morph is the different degrees of intra-morph compatibility in distinct morphs. Yet, there seems to be no strong pattern regarding which morph is more capable of intra-morph fertilization. Intra-morph compatibility can vary substantially, ranging from complete intra-morph incompatibility to full compatibility within the same morph. This variability has not only been observed between species within the same genus, e.g. *Primula* (Wedderburn and Richards 1990) and *Oxalis* (Zietsman *et al.* 2008), but also within the same species, e.g. *Primula merrilliana* (Shao *et al.* 2019).

These differences in intra-morph compatibility can be expected to affect reproduction and can be particularly apparent in the context of habitat fragmentation, leading to fewer suitable mates and disrupted pollination (Van Rossum *et al.* 2006). In *Primula veris*, which shows higher intra-morph compatibility in L-morphs than in S-morphs (Wedderburn and Richards 1990; Brys and Jacquemyn 2015), the dominance of L-morphs in a population was related to an overall higher seed set of the L-morph in small populations, but in large populations, the opposite pattern was observed, with S-morphs producing more seeds (Van Rossum *et al.* 2006). These findings indicate that due to their higher intra-morph compatibility, L-morphs can have reproductive advantages in fragmented habitats (Van Rossum *et al.* 2006). Similarly, in populations of *Primula sieboldii*, the mean fruit and seed set of L-morphs were higher, also likely influenced by the higher self-compatibility of L-morphs in this species (Matsumura and Washitani 2000).

Several studies show an overall dominance or deficit of one particular morph regardless of population size. A large-scale study on morph ratios in *Primula veris* found an overall excess of the S-morph across populations of different sizes, indicating a fitness advantage of the S-morph over the more self-compatible L-morph (Aavik *et al.* 2020). Similarly, Aronne *et al.* (2014) found S-morphs to be more abundant in *Primula palinuri*. In tristylous *Lythrum salicaria*, the S-morph is the least frequent morph (Andersson 1994). Generally, S-morphs produce less pollen than L-morphs, although the pollen grains in S-morphs tend to be larger (Ornduff 1980; Dulberger 1992). Furthermore, the pollen viability of different morphs can vary. For example, Aronne *et al.* (2021) showed that pollen of the S-morph of *Primula palinuri* had a significantly higher viability than that of the L-morph. In addition, S-morph pollen grains were less affected by elevated levels of humidity compared to L-morph pollen. The authors suggest that the higher viability of S-morph pollen may partly explain the dominance of S-morphs and that climate change may further amplify the reduced survival of pollen from L-morphs (Aronne *et al.* 2021).

### The impact of habitat loss and fragmentation on genetic diversity and differentiation

As a result of habitat loss and fragmentation, most plant populations become smaller and more isolated from each other (Young *et al.* 1996). Reduction in population size is one of the main factors influencing the genetic diversity of plant populations (Leimu *et al.* 2006). Small, isolated populations are more prone to genetic drift and may have undergone genetic bottlenecks following fragmentation events, and with smaller population size, there are fewer individuals for reproduction, which can result in higher inbreeding (Young *et al.* 1996; Leimu *et al.* 2010; Yuan *et al.* 2012). The negative effects of habitat fragmentation on plant genetic diversity and fitness are widely recognized, particularly in self-incompatible species (Leimu *et al.* 2006). Heterostylous self-incompatible species are thus particularly threatened because they depend on the availability of suitable morphs and on the abundance and diversity of pollinators. Numerous studies have indeed observed lower genetic diversity in smaller populations of heterostylous plants (Ness *et al.* 2010; Silva *et al.* 2014; Raabová *et al.* 2015). Morph ratio skewness further contributes to lower genetic diversity and higher inbreeding (Van Rossum and Triest 2006a; Meeus, Honnay, Brys, *et al.* 2012; Kaldra *et al.* 2023), although an association between genetic diversity and morph bias is not always found (Van Rossum and Triest 2006b). Populations skewed towards the L-morph have been commonly observed to have higher genetic diversity and lower inbreeding compared to populations skewed towards the S-morph (Van Rossum and Triest 2006b; Meeus, Honnay, Brys, *et al.* 2012; Kaldra *et al.* 2023). This has been attributed to the higher intra-morph self-compatibility in L-morphs of distylous plants (Wedderburn and Richards 1990), leading to more mating opportunities, especially in fragmented habitat conditions.

Besides the effect of variable degrees of intra-morph self-compatibility between morphs on the overall genetic diversity of heterostylous plant populations (Wedderburn and Richards 1990), differences in intra-morph compatibility can be expected to lead to differences in the within-population genetic diversity of different morphs as well as distinct morph-specific levels of genetic differentiation between populations. So far, no systematic differences between the genetic diversity of different morph types have been found (Van Rossum and Triest 2006a; Meeus, Honnay, Brys, *et al.* 2012; Kaldra *et al.* 2023). However, a study on *Primula veris* observed differences between morph types in response to landscape change. L-morphs showed higher differences in genetic diversity between regions that were differently affected by habitat fragmentation, while the genetic diversity of S-morphs remained more stable (Kaldra *et al.* 2023). In addition, the pairwise genetic differentiation (F_ST_) of S-morphs between populations was higher than that of L-morphs. These results indicate that there might be more gene flow between L-morphs due to their weaker intra-morph self-incompatibility (Kaldra *et al.* 2023).

Similarly to other plant species, the genetic diversity of heterostylous plant populations may be related to the location of a particular population within the distribution range of the species. In particular, populations located in the central part of the distribution range tend to have significantly higher genetic diversity than populations at the periphery, as shown in distylous *Abeliophyllum distichum* (Lee *et al.* 2022). In contrast, populations occurring further away from the main distribution range have lower genetic variation and are more genetically differentiated, suggesting reduced gene flow between these populations (Meeus, Honnay, and Jacquemyn 2012). In heterostylous plants, however, the disparities in the patterns of genetic diversity in relation to the location of populations may also be caused by the variation of intra-morph compatibility along the centre-periphery gradient. For example, in *Primula merrilliana*, peripheral populations contain more self-compatible and partly self-compatible individuals than central populations (Shao *et al.* 2019). This pattern aligns with Baker’s law, which states that colonization by self-compatible organisms is more likely to be successful than colonization by self-incompatible organisms (Baker 1955).

The timeframe during which the impact of habitat fragmentation on heterostylous plant populations becomes apparent in the patterns of genetic diversity and differentiation may substantially vary due to several reasons (Van Rossum and Triest 2007; Kaldra *et al.* 2023). First, the effect of habitat loss and fragmentation on genetic diversity may cause considerable time delays because of the long life span of the species (Epps and Keyghobadi 2015). It has been shown that the current genetic diversity in distylous *Primula veris* was indeed affected by past landscape characteristics rather than current environmental conditions (Reinula *et al.* 2021), indicating a delayed response to fragmentation (extinction debt, *sensu*  Helm *et al.* 2006). Extinction debt can also explain the more pronounced change of genetic diversity in seedlings compared to adults in *Primula elatior* (Van Rossum and Triest 2006a). Second, the ability to reproduce vegetatively may explain the lack of relationships between genetic diversity and fragmentation, with vegetatively reproducing species such as *Hottonia palustris* being less affected by habitat fragmentation than strictly sexually reproducing species (Vermeersch and Triest 2006). In addition, species with otherwise good dispersal abilities can maintain high genetic diversity regardless of fragmentation (Epps and Keyghobadi 2015). For example, in *Hedyotis chrysotricha,* seed dispersal through water contributes to maintaining functional connectivity between populations (Yuan *et al.* 2022).

### Evolutionary consequences of habitat fragmentation

Habitat fragmentation may cause evolutionary changes in several life history and reproductive traits, such as nectar production, seed dispersal, plant size, and mating strategy (Jacquemyn *et al.* 2012; Cheptou *et al.* 2017). In particular, the shift from outcrossing to selfing is recognized as one of the most common transitions in angiosperms (Cutter 2019) and has become increasingly likely in the context of human-induced fragmentation of habitats (Harder and Aizen 2010). Habitat fragmentation has been shown to shift mating patterns towards increased selfing as a result of different processes, which can also interact and thereby intensify the selection pressure on mating-related traits. These processes include increased isolation of populations and related scarcity of compatible mates due to the unbalanced sex ratios (Aguilar *et al.* 2008; Rodrigues *et al.* 2022) as well as fragmentation-induced decrease in the abundance and diversity of pollinators and resulting impacts on plant–pollinator interactions (Xiao *et al.* 2016; Millard *et al.* 2021). Furthermore, in the distylous *Primula oreodoxa*, decreased pollination has been associated with the loss of floral polymorphisms (Yuan *et al.* 2017). In heterostylous plants, a transition to self-compatibility is often accompanied by the shift from heterostyly to homostyly (Barrett 2019). The flowers of homostylous plants have anthers and stigma at the same height. The reduced distance between style and anthers can disrupt legitimate pollination through pollen deposition on the pollinator’s body (Keller *et al.* 2014; Brys and Jacquemyn 2020) and may lead to higher selfing rates (de Vos, Wüest, *et al.* 2014). Homostyles can be classified as either ‘long homostyles’, which have long styles and long anthers, or ‘short homostyles’, with short styles and short anthers. Long homostyles are more common than short homostyles (Charlesworth and Charlesworth 1979) and are considered as a ‘classic type of homostyly’ (Brys and Jacquemyn 2015). This transition has been observed in several populations of ancestrally heterostylous species (Crosby 1949; Curtis and Curtis 1985; Piper *et al.* 1986; Boyd *et al.* 1990; Li and Johnston 2011; Brys and Jacquemyn 2015; Barmentlo *et al.* 2018; Mora‐Carrera *et al.* 2023). The transition from heterostyly to homostyly in distylous plants is thought to be caused by recombination or mutations in the *S*-locus ‘supergene’, which consists of tightly linked genes that govern different traits related to heterostyly (Huu *et al.* 2016, 2020; Li *et al.* 2016). For example, mutation in the gene CYP734A50, one of the genes in the *S*-locus that controls style length and the female self-incompatibility type (Huu *et al.* 2021) and is present only in S-morphs (Huu *et al.* 2016), can result in a self-compatible long homostylous phenotype in *Primula* (Kappel *et al.* 2017; Mora‐Carrera *et al.* 2023). Furthermore, self-compatible heterostylous species are more likely to lose polymorphism than self-incompatible ones (Eckert and Barrett 1992).

The evolutionary trajectory for heterostylous species suffering from the lack or low levels of legitimate pollen caused by a low abundance of compatible mates or/and scarcity of pollinators can lead to the transition from heterostyly to homostyly (Costa *et al.* 2016; Yuan *et al.* 2017, 2023). However, the transition from heterostyly to homostyly may not be the only pathway as a consequence of limited pollination service. Variable levels of pollination in a plant species can lead to mixed mating, with either compatible heterostyly or plants with reduced herkogamy in monomorphic populations (Costa *et al.* 2016; Yuan *et al.* 2023). Disruption in pollinator services can lead to a shift in the reciprocal positioning of anthers and stigma, thereby reducing reproductive output (Brys and Jacquemyn 2015). A study on *Primula elatior* showed that the herkogamy was especially lower in S-morphs compared to the L-morphs in fragmented plant populations (Van Daele *et al.* 2024), indicating morph-specific responses to landscape change. However, there is currently little empirical evidence indicating that habitat fragmentation has driven such selection, as the loss of optimal reciprocity of reproductive organs may also reflect phenotypic plasticity and not evolutionary shifts (Jacquemyn *et al.* 2012).

To conclude, while being self-compatible can be advantageous in fragmented habitats, homostylous populations when compared to distylous populations of the same species, show higher selfing rates and significantly reduced genetic diversity (Yuan *et al.* 2017, 2023; Shao *et al.* 2019; Mora‐Carrera *et al.* 2023). Lower genetic diversity, in turn, is related to a decrease in the adaptive potential of plants (de Vos, Hughes, *et al.* 2014; Wang *et al.* 2021), which can make them more vulnerable to environmental changes and reduce their long-term viability (Leimu *et al.* 2010). Nevertheless, large variations in self-incompatibility and transitions from heterostyly to homostyly are thought to be the main drivers of rapid speciation (He *et al.* 2022).

## Conservation implications

Human-induced habitat loss and fragmentation lead to smaller and more isolated populations of plant species, with negative consequences for populations (e.g. Endels *et al.* 2007). All animal-pollinated species are susceptible to the negative effects of reduced population size and lower connectivity between populations—a process magnified by the decline of pollinators (Rodger *et al.* 2021). The heterostylous mating pattern introduces additional threats, such as skewed morph frequencies and lower availability of suitable mates in small and fragmented populations. Hence, protecting and maintaining a sufficient area of suitable habitats to support viable populations are essential for the short-term survival of heterostylous species and for enabling species to respond and adapt to landscape and climatic changes in the long term (Shao *et al.* 2015). As heterostyly is an adaptation to animal-pollination, it is also highly vital to support pollinators and their habitats to ensure the well-being of heterostylous species. Maintaining and creating landscape elements such as hedges, tree rows, ditch banks and road verges between habitat patches may support the movement of pollinators between spatially isolated patches of habitats. This, in turn, increases the probability of pollen dispersal between plant populations located on these isolated habitat patches, as was demonstrated in fragmented populations of *Primula vulgaris* (Van Geert *et al.* 2010), thereby also enlarging the abundance of legitimate mates. However, another study on *Primula vulgaris* showed that hedgerow networks are not the best elements in promoting gene flow and instead can even impede it (Campagne *et al.* 2009), most probably by acting as a barrier for some important pollinator groups. Thus, when choosing landscape elements for promoting connectivity, careful consideration should be given to the target species and the surrounding landscape.

Recognized strategies for conserving and managing severely fragmented plant populations include demographic interventions involving the introduction of individuals into existing populations. Specifically for heterostylous species, introducing individuals of the underrepresented or missing morph types may be very effective (Endels *et al.* 2002). If a small heterostylous self-incompatible population consists of only one morph type, the effective population size (N_e_) is essentially 0, and in fragmented habitats, pollen dispersal from neighbouring populations can also be restricted. Thus, introducing individuals of the opposite morph type can help restore legitimate pollination and increase successful reproduction (Endels *et al.* 2002). However, populations of *Primula vulgaris* managed by augmenting the populations with individuals from other populations unexpectedly showed signs of outbreeding depression (Barmentlo *et al.* 2018), although the overall fitness increased. Outbreeding depression, though rare (Frankham 2015), can reduce offspring fitness when ecologically and genetically distant gene pools mix, leading to lower local adaptation (Barmentlo *et al.* 2018). Hence, when introducing new individuals, including the missing morphs, to a population, the genetic distance of the populations should be considered whenever possible.

All conservation activities should be accompanied by monitoring to assess the effectiveness of the conservation measures. For heterostylous species, in addition to assessing traditional indicators of conservation success, such as population size, survival, seedling recruitment, reproductive output, and population growth rate, it is crucial to include the recording of morph frequencies. This can provide additional valuable information about the well-being and sustainability of the plant populations, particularly for heterostylous plants, where maintenance of balanced morph ratios is essential for population reproduction and long-term survival.

## Knowledge gaps

Despite the possible severe negative consequences of fragmentation on heterostylous plants, there are still relatively few studies systematically addressing the short- and long-term effects of habitat loss and decreased connectivity on these species. The current review shows that most of the studies have focused on representatives from the *Primula* genus. Specifically, out of the 149 studies explored in this review, almost 1/3 examined a representative of *Primula*. However, among the 28 families where the heterostylous breeding system occurs, many have gained no attention at all. While knowledge based on information from *P. veris, P. vulgaris,* and other *Primula* species (a genus where heterostyly is particularly common) has provided invaluable insights into the phylogenetic background and molecular mechanisms governing heterostyly, the scarce evidence from other plant families does not allow making broad generalizations about the possible effects of habitat loss and other factors of global change on heterostylous plants.

A simultaneous study on different heterostylous species in the same habitats would produce novel insights into the generality of morph-ratio changes in response to habitat loss. If similar patterns emerge in different heterostylous species with similar ecological requirements in response to fragmentation, it can indeed be concluded that heterostylous species require specific attention to diminish the negative impacts of this process. Furthermore, it would allow morph ratio patterns to serve as a proxy indicator for assessing the extent of habitat loss on plant population viability. While the first attempts have been made to quantify the effects of landscape characteristics on morph ratios in *P. veris* (Aavik *et al.* 2020), it is important to gain more insights into how morph frequencies respond to landscape changes (e.g. in continuous and fragmented landscape settings, in forest landscapes and urban areas) and how it might be affected by the environmental gradient. Additionally, long-term morph frequency monitoring in the same populations would provide insight into fluctuations of morph ratios over the years and shed light on the potential impact of extreme climate events (e.g. drought and flooding) on morph balance. Currently, only one study has explored morph ratio changes over time (Endels *et al.* 2002).

Knowledge regarding the impact of habitat loss and fragmentation on the genetic diversity and overall viability of heterostylous plant populations remains limited despite their higher vulnerability to recent land use changes compared to self-compatible species and species not depending on animal-pollination (Meeus, Honnay, Brys, *et al.* 2012; Kaldra *et al.* 2023). More studies on other heterostylous species are needed that simultaneously assess morph ratios and genetic diversity. If a similar correlation is observed in other species, repeated monitoring of morph frequencies can provide valuable insights into the long-term sustainability of populations. There are also relatively few studies exploring the potential for genetic rescue in endangered heterostylous populations. More experimental and practical conservation actions are needed to understand how to effectively support and maintain these populations before it is too late.

A better understanding of the varying degrees of intra-morph compatibility of distinct morphs is vital for predicting the short- and long-term effects of habitat loss and fragmentation on heterostylous species. Previous research has already shown that heterostylous species exhibit a wide range of within-morph self-compatibilities, ranging from complete self-incompatibility to relatively high self-compatibility within the same morph (Wedderburn and Richards 1990; Zietsman *et al.* 2008). Different levels of (intra-morph) self-compatibility can lead to varying responses when populations experience reductions in population size or imbalances in mate availabilities. However, the existing knowledge is fragmented, and there is significant room for improvement through integrated observational and experimental studies to assess the generality of the patterns of intra-morph and self-compatibility in heterostylous plants. Furthermore, there is evidence that skewed morph ratios may intensify hybridization and introgression events between species within the same genera (Keller *et al.* 2016). This can happen, for example, when a small population with unbalanced morph frequency may ‘compensate’ for the scarcity of compatible conspecific mates by receiving pollen from the compatible morph of the neighbouring relative species with a large population size (Keller *et al.* 2016). Thus, the impacts of fragmentation on heterostylous plants may extend beyond the population- and species-level consequences.

Although some studies report the evolution of homostyly in response to the scarcity of mates and lack of pollinators (e.g. Barmentlo *et al.* 2018; Mora‐Carrera *et al.* 2023), we cannot be sure how widespread this phenomenon is and whether this trend can be expected in other heterostylous species threatened by fragmentation and pollination limitations. More extensive, large-scale monitoring should be carried out across different heterostylous taxa to understand the generalizability of possible shifts in mating patterns. This knowledge could complement recent advances in understanding the molecular mechanisms underlying mating pattern shifts in heterostylous species (Kappel *et al.* 2017; Shore *et al.* 2019; Huu *et al.* 2020; Potente *et al.* 2022; Fawcett *et al.* 2023).

Biodiversity is affected by both land-use change and shifts in climatic conditions, yet we are only starting to comprehend the interacting effects of these variables on biodiversity (Outhwaite *et al.* 2022; Suggitt *et al.* 2023). Likewise, there is limited evidence regarding the combined effects of habitat fragmentation and climate change on heterostylous plants. A recent study shows that habitat fragmentation can disrupt climate adaptation and resilience in heterostylous *Primula elatior* (Van Daele *et al.* 2024). Similarly, a study on *Primula veris* showed that higher precipitation contributes to the prevalence of S-morphs, and increasing proportions of cropland and built-up areas in the surroundings of populations lead to more biased morph frequencies (Aavik *et al.* 2025). These studies emphasize the importance of considering the impacts of both climate and landscape change effects, as these factors have the potential to amplify each other’s adverse effects.

## Conclusions

Our review shows that habitat loss and fragmentation can lead to various short- and long-term consequences for heterostylous plant species. All animal-pollinated species are susceptible to the negative effects of reduced population size and lower connectivity between populations—a process magnified for these plants by the ongoing loss of pollinators (Rodger *et al.* 2021). However, plants with heterostylous mating systems are particularly vulnerable to both habitat loss and shifts in pollinator abundance because they require suitable morphs to be present for reproduction. Reductions in population size lead to skewed morph frequencies, and this way lowers the availability of suitable mates. Intra-morph and self-incompatibility make them highly reliable for pollinators. Hence, these general and heterostyly-specific effects can amplify each other’s negative effects and make heterostylous species even more vulnerable to landscape changes. As the fragmentation of natural and semi-natural habitats remains a persistent threat to biodiversity, an improved understanding of its effects is crucial to prevent further losses of vulnerable species (Haddad *et al.* 2015).

## Supplementary Material

plaf016_suppl_Supplementary_Material

## Data Availability

Data used for meta-analysis is available in the supplementary materials. Supplementary information contains a table with the literature search results, a PRISMA protocol conducted on the literature and the data used for the meta-analysis.
