# Supplementary material for: Heterostylous plants in an era of global change: a review on the consequences of habitat loss and fragmentation
Source: AoB Plants. 2025 Mar 22;17(4):plaf016. doi: 10.1093/aobpla/plaf016 (PMC12285733; doi:10.1093/aobpla/plaf016)
Supplement: plaf016_suppl_Supplementary_Material [file plaf016_suppl_supplementary_material.zip › Supplementary Data/Supplementary_PRISMA.pdf]

# Heterostylous plants in an era of global change: a review on the consequences of habitat loss and fragmentation

Marianne Kivastik[1*], Sílvia Castro[2], Elena Conti[3], Hans Jacquemyn[4], Barbara Keller[3], Attila Lengyel[5,6], Michael Lenhard[7], Zuzana Münzbergová[8], Iris Reinula[1], Bojana Stojanova[9], Sabrina Träger[10,11], Mari-Liis Viljur[1], Tsipe Aavik[1]

Supplementary materials

PRISMA 2020 flow diagram for new systematic reviews which included searches of databases and registers only (Page *et al.* 2021) with detailed descriptions of the steps implemented.

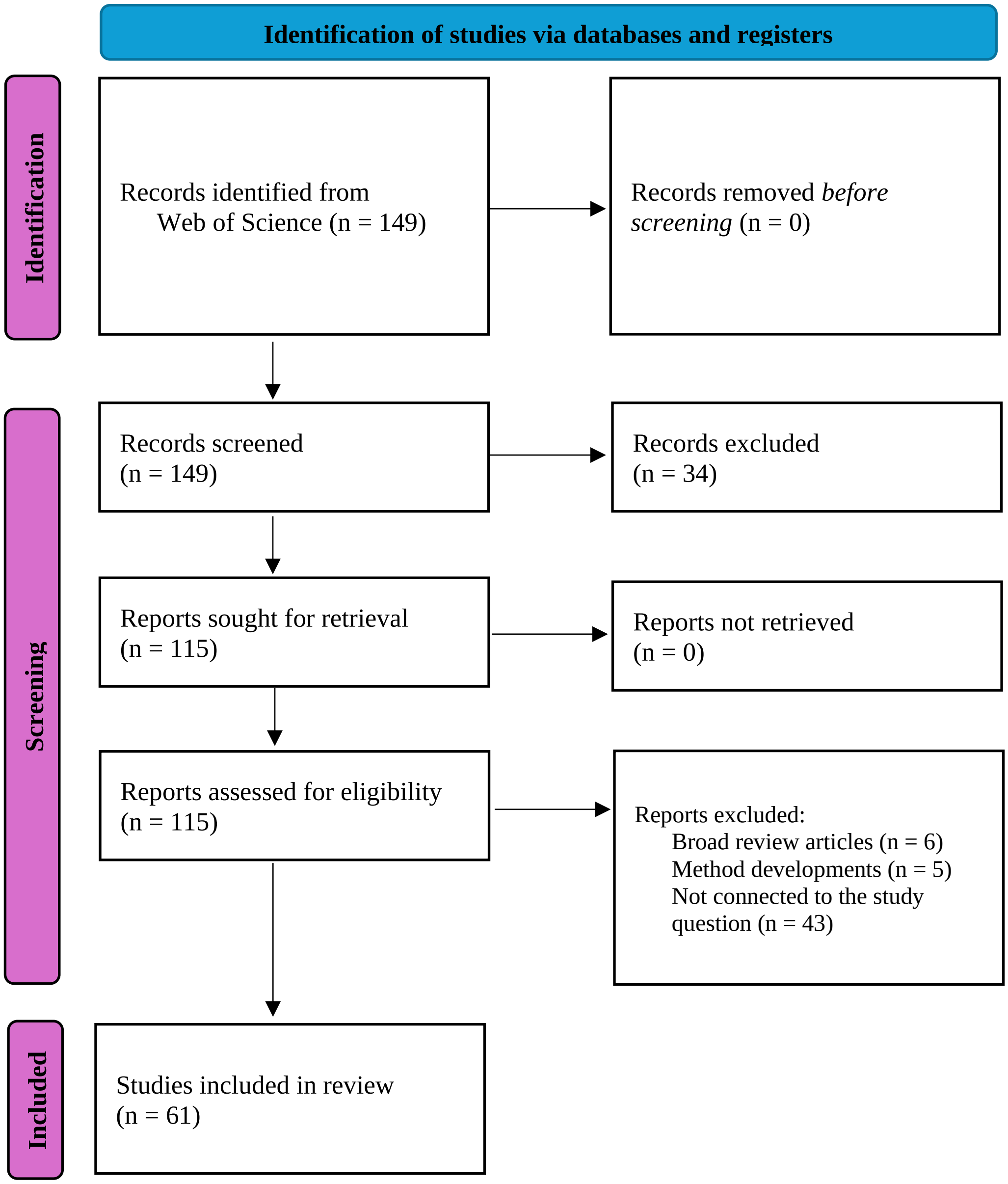

We conducted the PRISMA protocol (Page *et al.* 2021) on the literature search results to identify suitable studies. There was no need to remove any records before the screening, as the search was done only on one database (Web of Science), therefore, no duplicates were found, and no automation tools were used. All search results were initially screened by the titles and abstracts before a more detailed evaluation. This screening excluded records that were:

(1) not written in English (only English abstracts available), n = 5;

(2) the word string led to a different meaning. For example, for the keyword 'fragment*', a few articles discussed DNA fragments rather than habitat fragments. Similarly, for the keyword 'heterostyl*', results included mentions of an animal species, such as the fly *Heterostylodes macrurus* (Anthomyidae), n = 9;

(3) papers were of other (polymorphic) species and not heterostylous species (e.g., style-dimorphic *Narcissus* species), n = 20.

This screening resulted in the exclusion of 34 articles. All remaining 115 articles were then further assessed for their suitability. Here, articles were excluded when they were not suitable content-wise, such as:

(1) broad review articles, n = 6;

(2) method developments or computational models (e.g., microsatellite markers), n = 5;

(3) otherwise not connected to the study question (e.g., descriptions of species in general or determination of heterostyly in a particular species, relevant keywords came up only in the references, but not the actual article text etc.), n = 43.

This excluded a further 54 articles and resulted in a total of 61 articles relevant to the study question.

## Literature cited

Page MJ, McKenzie JE, Bossuyt PM, *et al.* 2021. The PRISMA 2020 statement: an updated guideline for reporting systematic reviews. *BMJ*: n71.
